# ﻿First complete mitochondrial genome of the tribe Coccini (Hemiptera, Coccomorpha, Coccidae) and its phylogenetic implications

**DOI:** 10.3897/zookeys.1180.109116

**Published:** 2023-09-26

**Authors:** Yun-Feng Hou, Jiu-Feng Wei, Tian-You Zhao, Cai-Feng Li, Fang Wang

**Affiliations:** 1 Ministry of Education Key Laboratory of Molecular and Cellular Biology, Hebei Collaborative Innovation Center for Eco-Environment, Hebei Key Laboratory of Animal Physiology, Biochemistry and Molecular Biology, College of Life Sciences, Hebei Normal University, Shijiazhuang, Hebei, 050024, China Hebei Normal University Shijiazhuang China; 2 College of Plant Protection, Shanxi Agricultural University, Jinzhong, Shanxi, 030801, China Shanxi Agricultural University Jinzhong China; 3 Department of Entomology and MOA Key Lab of Pest Monitoring and Green Management, College of Plant Protection, China Agricultural University, Beijing, 100193, China China Agricultural University Beijing China

**Keywords:** *Coccushesperidum*, comparative mitochondrial genomics, mitogenome, phylogenetic analysis

## Abstract

Soft scale insects (Hemiptera, Coccidae) are important pests of various agricultural and horticultural crops and ornamental plants. They have negative impacts on agriculture and forestry. The tribe Coccini represents one of the most ancient evolutionary lineages of soft scale insects. However, no complete Coccini mitochondrial genome (mitogenome) is available in public databases. Here, we described the complete mitogenome of *Coccushesperidum* L., 1758. The 15,566 bp mitogenome of *C.hesperidum* had a high A+T content (83.4%) and contained a typical set of 37 genes, with 13 protein-coding genes (PCGs), 22 transfer RNA genes (tRNAs) and two ribosomal RNA genes (rRNAs). Only seven tRNAs had the typical clover-leaf secondary structure and the remaining tRNAs lacked the DHU arm, TψC arm or both. Moreover, a comparative analysis of all reported scale insect mitogenomes from GenBank database was performed. The mitogenomes of scale insects showed high similarities in base composition and A+T content. Additionally, our phylogenetic analysis confirmed the monophyly of Coccomorpha and revealed that the archaeococcoids were the most basal lineage within Coccomorpha, while *Ericeruspela* and *Didesmococcuskoreanus*, belonging to Coccidae, were often mixed with Aclerdidae, making Coccidae a paraphyletic group. These findings expand the mitogenome database of scale insects and provide new insights on mitogenome evolution for future studies across different insect groups.

## ﻿Introduction

Scale insects (Coccomorpha) belong to the suborder Sternorrhyncha and include more than 8500 species worldwide (Gullan and Martin 2009; García Morales et al. 2016). The family Coccidae, known as soft scales, is the third largest within Coccomorpha after Diaspididae (armoured scales) and Pseudococcidae (mealybugs) (Wang and Feng 2012). Soft scales have distinct piercing-sucking mouthparts and the dorsum of most species is generally covered with various waxy substances or is convex, forming a nearly rounded body, with substantial morphological diversity (Gullan and Martin 2009; Lu et al. 2023). Many species are important economic pests of agricultural and horticultural crops and ornamental plants, causing damage by the extraction of nutrients and water, injection of salivary toxins or transmission of plant virus diseases (Kondo and Watson 2022), such as *Ceroplastesrusci*, *C.japonicus* and *Parasaissetianigra* which species have high reproductive rates (Lin et al. 2017a; Choi and Lee 2020; Shan et al. 2023). However, some species are beneficial to humans, such as *Ericeruspela*, whose wax provides an important raw material for many industries (Kondo and Gullan 2022; An et al. 2023). Further, the soft scale, *Pulvinariellamesembryanthemi* is being considered as a biocontrol agent for weedy ice plants (Kondo and Gullan 2022).

*Coccus* is the oldest genus within Coccidae, proposed by Linnaeus in 1758 with *Coccushesperidum* L. as its type species; it belongs to the tribe Coccini and subfamily Coccinae (Hodgson 1994). *Coccushesperidum* contains two subspecies, *C.hesperidumhesperidum* with a widespread distribution and *C.hesperidumjavanensis* located only in Java Island of Indonesia, the former is commonly referred to as the brown soft scale (García Morales et al. 2016). *Coccushesperidum* is an economically important pest and highly polyphagous, with reports of significant damage to plants in approximately 417 genera from 138 families across 177 countries (García Morales et al. 2016; Lin et al. 2017b; Villanueva et al. 2020; Kondo et al. 2022). This pest damages host plants by sucking their phloem sap, affecting photosynthesis and plant growth and secreting honeydew, often inducing growth of sooty moulds (Aliakbarpour et al. 2010; Choi and Lee 2018; Kondo et al. 2022). In the last few decades, most research, focused on the identification, morphology, biology and ecology of *C.hesperidum*, has aimed to provide a basis for controlling and preventing the spread of this species (Kapranas et al. 2009; Aliakbarpour et al. 2010; Golan et al. 2013; Lin et al. 2013; Fan et al. 2014).

Mitochondria are organelles involved in energy metabolism in eukaryotic cells (Cameron 2014). The mitochondrial genome (mitogenome) is characterised by maternal inheritance, low recombination rates and high mutation rates and it has been used as a molecular marker in phylogeny, biogeography and other evolutionary studies (Cameron 2014; Lu et al. 2020; Xu et al. 2023) in different insect groups, such as in Hymenoptera, Hemiptera and Neuroptera (Cui et al. 2013; Tang et al. 2019; Jiang et al. 2022). However, only 16 coccid mitogenomes have been reported to date, including seven Coccidae mitogenomes, two each of Aclerdidae and Eriococcidae, one each of Pseudococcidae, Matsucoccidae, Cerococcidae, Kerriidae and Monophlebidae (Deng et al. 2019; Song et al. 2019; Liu et al. 2020; Xu and Wu 2022; An et al. 2023; Lu et al. 2023; Xu et al. 2023). Compared to more than 8500 scale insect species recorded globally, the available mitogenomes are highly limited. Thus, mitogenomes for sequencing of scale insects is an important aim for understanding the phylogenetic relationships of Coccomorpha and even Sternorrhyncha.

Mitogenomes of *Coccus* or even Coccini have not been reported to date. Thus, we sequenced and analysed the detailed features of the complete mitogenome of *C.hesperidum*. Then, we compared the mitogenome characteristics for all reported scale insects mitogenomes. In addition, we investigated the mitogenome phylogeny of Sternorrhyncha, to assess the phylogenetic position of *C.hesperidum*. These findings expand the mitogenome database of scale insects and provide a significant basis for future studies of the phylogeny and evolution of Hemiptera.

## ﻿Materials and methods

### ﻿Sampling, DNA extraction and sequencing

*Coccushesperidum* was collected from *Radermacherasinica* (Bignoniaceae) on 19 May 2019, in Shijiazhuang (37°59'58"N, 114°30'59"E), Hebei Province, China. Then, the scale insects were stored in absolute ethanol at -80 °C. The samples were identified, based on morphological characteristics and molecular identification. For each specimen, total DNA was extracted from the body using a QIAamp DNA Mini Kit (Qiagen, Hilden, Germany) according to the extraction protocol. The concentration and quality of DNA were determined by a NanoDrop 2000 spectrophotometer (Thermo Fisher Scientific, Waltham, MA, USA) and 1% agarose gel electrophoresis and samples were then stored at -20 °C. A genomic DNA library was constructed with 1 µg of DNA that was fragmented into 300–500 bp fragments using the Covaris ME220 Focused Ultrasonicator (Thermo Fisher Scientific). After end-repair, “A” tailing, adapter ligation, purification and PCR amplification, the fragments were sequenced using the paired-end 150 sequencing method on the Illumina NovaSeq 6000 platform by Novogene Bioinformatics Technology Co., Ltd. (Tianjin, China).

### ﻿Mitogenome assembly and annotation

Approximately 30 Gb of clean data were obtained after filtering the raw data by removing adapter sequences and low-quality reads (quality value < 20). The complete mitogenome was assembled by Novoplasty (Dierckxsens et al. 2017) annotated using the MITOS Web Server (Donath et al. 2019) and MitoZ v.2.4 (Meng et al. 2019) and subsequently manually corrected. The boundaries of protein-coding genes (PCGs) were confirmed manually using the ORF finder in the National Center for Biotechnology Information (NCBI). The final mitogenome map was exported by MitoZ v.2.4.

### ﻿Gene metrics and comparative mitogenomes

The secondary structures of transfer RNA genes (tRNAs) were predicted using the ViennaRNA module (Gruber et al. 2015) built in MITOS2 (Bernt et al. 2013) and RNAstructure v.6.3 (Reuter and Mathews 2010). The base composition, amino acid usage and relative synonymous codon usage (RSCU) were calculated using MEGA X (Kumar et al. 2018) and a Python script that refers to the CAI module (Lee 2018). To better understand the codon usage bias of the mitogenome of *C.hesperidum*, the following formulae were used: AT skew = (A–T)/(A + T); GC skew = (G–C)/(G + C) (Perna and Kocher 1995). Furthermore, the synonymous substitution rate (Ks) and the non-synonymous substitution rate (Ka) for each PCG were calculated using DnaSP v.6.12.03 (Rozas et al. 2017). Additionally, a comparative analysis of reported scale insect mitogenomes was performed in terms of base composition, RSCU, AT/GC skew and Ka/Ks ratio.

### ﻿Phylogenetic analysis

For phylogenetic analysis, the newly-obtained mitogenome data for *Coccushesperidum* in this study and 51 other representative Sternorrhyncha species from the GenBank database were sampled (Suppl. material 1: table S1). The ingroup taxa included Psylloidea (psyllids), Aleyrodoidea (whiteflies), Aphidomorpha (aphids) and Coccomorpha (scale insects). Amongst them, psyllids and aphids contained representative species from almost every family, whiteflies contained species from the two subfamilies Aleyrodinae and Aleurodicinae and scale insects included all reported mitogenomes species, except *Drosichacorpulenta* (incomplete numbers of PCGs). *Cryptotympanaatrata* (Auchenorrhyncha, Cicadoidea) and *Populiceruspopuli* (Auchenorrhyncha, Membracoidea) were selected as outgroup taxa to root the Maximum Likelihood (ML) and Bayesian Inference (BI) trees. We used two different datasets in our phylogenetic analysis with different combinations of the nucleotide sequences of 13 PCGs and two rRNAs, as well as amino acid sequences of the protein coding genes: 1) amino acid sequences of the 13 PCGs (PCGAA) and 2) complete nucleotide sequences of 13 PCGs and two rRNAs (PCG123rRNA), which were extracted using PhyloSuite v.1.2.2 (Zhang et al. 2020). Each dataset was aligned separately using MAFFT v.7.313 (Katoh and Standley 2013). Ambiguous sites and poorly-aligned positions were removed using trimAl v.1.2, where the automated trimming parameter was set to “automated 1” and other parameters with default settings (Capella-Gutiérrez et al. 2009) and the individual gene regions were concatenated in PhyloSuite v.1.2.2. The substitution models were estimated using PartitionFinder 2 (Cognato and Vogler 2001), based on the Bayesian Information Criterion (BIC). The ML analyses were conducted using IQ-TREE v.2.1.2 with 1,000 replicates (Minh et al. 2020) and the BI trees were generated using MrBayes 3.2.6 (Ronquist et al. 2012), with four independent Markov Chain Monte Carlo runs of 2,000,000 generations. The resulting phylogenetic trees were visualised using FigTree v.1.4.4 (Rambaut 2018) and the iTOL online webserver.

## ﻿Results

### ﻿Genome organisation

**Figure 1.**
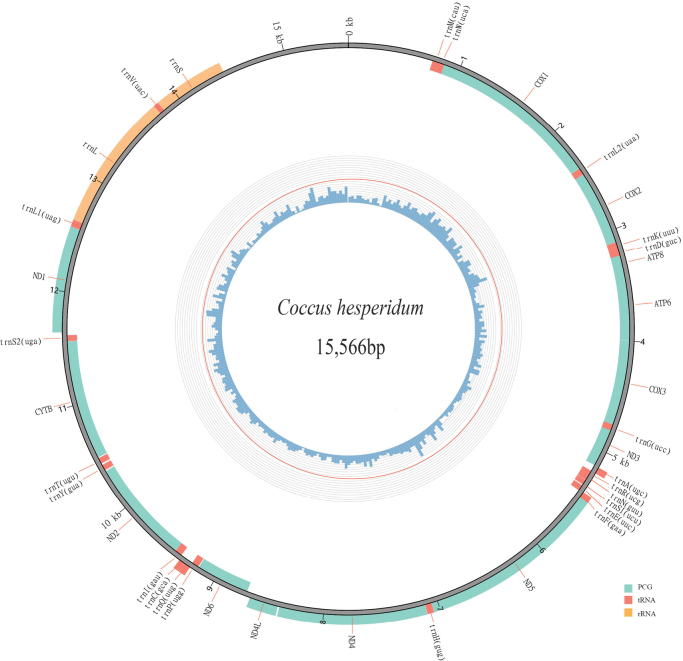
Circular mitochondrial genome map of *Coccushesperidum*. Genes inside the map are on the forward strand, genes outside are on the reverse strand. The interior histogram shows the GC content calculated in every 50-site window.

The complete mitogenome sequence of *Coccushesperidum* was assembled into a single contig of 15,566 bp in length, including 13 PCGs, 22 tRNAs and two rRNAs, amongst which 24 genes (9 PCGs and 15 tRNAs) were encoded on the forward strand (+), while the other four PCGs, seven tRNAs and two rRNAs were on the reverse strand (-) (Table 1 and Fig. 1). The mitogenome sequence has been deposited in GenBank (accession number: OR167606). The overall base composition of the mitogenome sequence was 50.9%A, 32.5% T, 10.9% C, 5.8% G, with a strong bias towards A+T (83.4%). The A+T contents of PCGs, tRNAs, and rRNAs of *C.hesperidum* were 82.1%, 87.8% and 86.0%, respectively. Thirteen regions of gene overlap between adjacent genes were detected in the mitogenome of *C.hesperidum* and the two longest overlaps were located between *trnW*-*COX1* and *trnE*-*trnF* with a length of 10 bp. In addition, there were twelve intergenic spacers and the longest was found between *ND4L* and *ND6* with 40 bp (Table 1).

**Table 1.** Mitochondrial genome characteristics of *Coccushesperidum*.

Gene	Location	Size (bp)	Strand	Start codon	Stop codon	Anticodon	Intergenic length
*trnM*	753–818	66	+			CAU	
*trnW*	810–870	61	+			UCA	-9
*COX1*	861–2387	1527	+	ATA	TAA	–	-10
*trnL_2_*	2388–2449	62	+			UAA	0
*COX2*	2450–3113	664	+	ATA	T	–	0
*trnK*	3114–3183	70	+			UUU	0
*trnD*	3180–3244	65	+			GUC	-4
*ATP8*	3238–3378	141	+	ATT	TAA	–	-7
*ATP6*	3372–3989	618	+	ATG	TAA	–	-7
*COX3*	3991–4752	762	+	ATG	TAA	–	1
*trnG*	4752–4809	58	+			UCC	-1
*ND3*	4807–5145	339	+	ATA	TAA	–	-3
*trnA*	5146–5203	58	–			UGC	0
trn*R*	5202–5249	48	+			UCG	-2
*trnN*	5241–5294	54	+			GUU	-9
*trnS_1_*	5296–5343	48	+			UCU	1
*trnE*	5352–5405	54	+			UUC	8
*trnF*	5396–5452	57	–			GAA	-10
*ND5*	5451–7055	1605	–	ATT	TAA	–	-2
*trnH*	7056–7112	57	–			GUG	0
*ND4*	7114–8388	1275	–	ATT	TAA	–	1
*ND4L*	8401–8655	255	–	ATT	TAA	–	12
*ND6*	8696–9172	477	+	ATT	TAG	–	40
*trnP*	9179–9241	63	+			UGG	6
*trnQ*	9238–9295	58	–			UUG	-4
*trnC*	9295–9349	55	–			GCA	-1
*trnI*	9359–9423	65	+			GAU	-10
*ND2*	9424–10,362	939	+	ATT	TAA	–	0
*trnY*	10,373–10,425	53	+			GUA	10
*trnT*	10,440–10,493	54	+			UGU	14
*CYTB*	10,501–11,568	1068	+	ATA	TAA	–	7
*trnS_2_*	11,570–11,622	53	+			UGA	1
*ND1*	11,647–12,552	906	–	ATT	TAA	–	24
*trnL_1_*	12,553–12,615	63	–			UAG	0
16S rRNA	12,616–13,789	1174	–			–	0
*trnV*	13,790–13,835	46	–			UAC	0
12S rRNA	13,836–14,447	612	–			–	0

### ﻿Protein-coding genes

**Figure 2.**
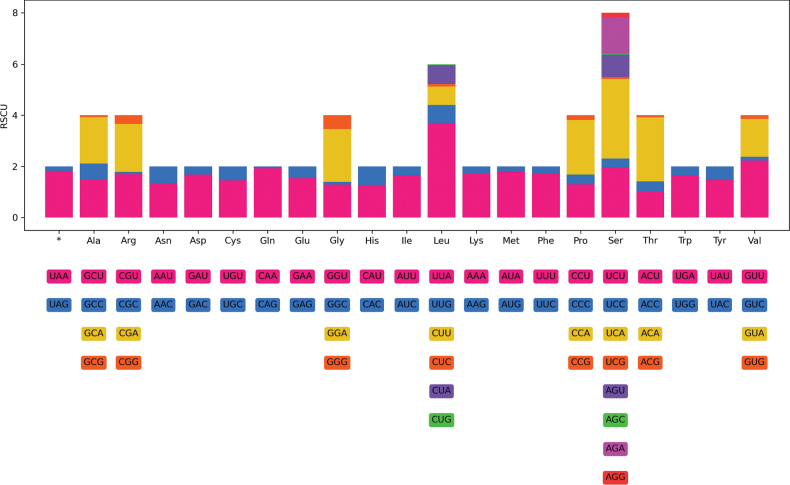
The relative synonymous codon usage (RSCU) of protein-coding genes (PCGs) in *Coccushesperidum* mitochondrial genome.

The total length of 13 PCGs was 10,575 bp, accounting for 67.9% of the complete mitogenome length of *Coccushesperidum*. Mostly, PCGs reside on the forward strand, except *ND1*, *ND4*, *ND4L* and *ND5*, which were on the reverse strand. The AT/GC skew values for the 13 PCGs were -0.48 to 0.26 and -0.54 to 0.66, respectively (Table 2). All PCGs in the *C.hesperidum* mitogenome used ATN as the initiation codon, of which four genes (*COX1*, *COX2*, *ND3* and *CYTB*) started with the codon ATA, seven genes (*ATP8*, *ND6*, *ND2*, *ND5*, *ND4*, *ND4L* and *ND1*) started with ATT and the other genes (*ATP6* and *COX3*) started with ATG. Moreover, eleven PCGs terminated with the codon TAA, except *ND6* with TAG as the stop codon, while *COX2* ended with a single T. This is common in other hemipteran mitogenomes, where these incomplete stop codons form the complete stop codon TAA by the addition of “A” to the 3’ end of the mRNA, thus terminating transcription through polyadenylation (Ojala et al. 1981; Xu et al. 2019; Lu et al. 2023). The RSCU values of PCGs are illustrated in Fig. 2 and Table 3. The highest RSCU value was 3.696 for the codon TTA (Leu) and the lowest value was 0.045 for CTG (Leu).

**Table 2.** Nucleotide compositions and AT/GC skews in mitochondrial genome of *Coccushesperidum*.

Gene	Nucleotide frequency				A+T(%)	AT-skew	GC-skew
	A(%)	T(%)	G(%)	C(%)			
*COX1*	40.0	35.8	8.8	15.3	75.8	0.06	-0.27
*COX2*	48.9	29.7	7.8	13.6	78.6	0.24	-0.27
*COX3*	45.0	37.0	5.4	12.6	82	0.10	-0.40
*ATP8*	55.3	36.2	2.8	5.7	91.5	0.21	-0.34
*ATP6*	49.8	34.0	3.7	12.5	83.8	0.19	-0.54
*ND1*	25.3	56.6	12.1	6.0	81.9	-0.38	0.34
*ND2*	50.7	34.0	5.0	10.3	84.7	0.20	-0.35
*ND3*	54.3	32.2	3.8	9.7	86.5	0.26	-0.44
*ND4*	22.2	62.4	10.2	5.2	84.6	-0.48	0.32
*ND4L*	23.9	64.3	9.8	2.0	88.2	-0.46	0.66
*ND5*	23.5	60.2	10.0	6.2	83.7	-0.44	0.23
*ND6*	55.3	32.3	3.1	9.2	87.6	0.26	-0.50
*CYTB*	44.1	34.6	7.4	13.9	78.7	0.12	-0.31
22 tRNAs	46.7	41.1	6.2	6.0	87.8	0.06	0.02
*rrnL*	38.3	49.2	8.3	4.1	87.56	-0.12	0.34
*rrnS*	36.4	46.60	10.9	6.0	83.01	-0.12	0.29
2 rRNAs	37.7	48.3	9.2	4.8	86.0	-0.14	0.31
Total	50.9	32.5	5.8	10.9	83.4	0.22	-0.31

**Table 3.** The relative synonymous codon usage (RSCU) of *Coccushesperidum*.

AA	Codon	Count	RSCU	AA	Codon	Count	RSCU	AA	Codon	Count	RSCU	AA	Codon	Count	RSCU
Leu	TTA	247	3.696	Ile	ATT	324	1.653	His	CAC	14	0.718	Lys	AAG	29	0.267
Ser	TCA	116	3.114	Trp	TGA	47	1.649	Leu	TTG	47	0.703	Met	ATG	52	0.202
Thr	ACA	84	2.489	Glu	GAA	48	1.548	Asn	AAC	96	0.671	Pro	CCG	3	0.188
Val	GTT	91	2.233	Ala	GCT	10	1.509	Ala	GCC	4	0.604	*	TAG	1	0.167
Pro	CCA	34	2.125	Val	GTA	60	1.472	Gly	GGG	10	0.541	Ser	AGG	6	0.161
Gly	GGA	38	2.054	Tyr	TAT	130	1.469	Cys	TGC	4	0.533	Val	GTC	6	0.147
Ser	TCT	73	1.96	Cys	TGT	11	1.467	Tyr	TAC	47	0.531	Val	GTG	6	0.147
Gln	CAA	30	1.935	Ser	AGA	53	1.423	Glu	GAG	14	0.452	Gly	GGC	2	0.108
Arg	CGA	11	1.872	Asn	AAT	190	1.329	Thr	ACC	13	0.385	Leu	CTC	7	0.105
*	TAA	11	1.833	Pro	CCT	21	1.312	Pro	CCC	6	0.375	Thr	ACG	3	0.089
Ala	GCA	12	1.811	Gly	GGT	24	1.297	Trp	TGG	10	0.351	Arg	CGC	0.5	0.085
Met	ATA	463	1.798	His	CAT	25	1.282	Ser	TCC	13	0.349	Ser	TCG	3	0.081
Lys	AAA	188	1.733	Thr	ACT	35	1.037	Ile	ATC	68	0.347	Ala	GCG	0.5	0.075
Phe	TTT	413	1.721	Ser	AGT	32	0.859	Arg	CGG	2	0.34	Gln	CAG	1	0.065
Arg	CGT	10	1.702	Leu	CTT	49	0.733	Asp	GAC	9	0.31	Ser	AGC	2	0.054
Asp	GAT	49	1.69	Leu	CTA	48	0.718	Phe	TTC	67	0.279	Leu	CTG	3	0.045

The most frequently used codons were ATA, TTT and ATT (Suppl. material 1: table S2). Moreover, RSCU illustrated the over-utilisation of A or T nucleotides in the third codon position. For example, in synonymous codons encoding Met, the ATA codon was used 463 times with an RSCU of 1.798, while ATG was only used 52 times with an RSCU of 0.202; for Lys, the AAA codon was used 188 times (RSCU = 1.733) and the AAG codon was used only 29 times (RSCU = 0.267).

### ﻿Transfer RNA genes and ribosomal RNA genes

In total, 22 tRNA genes with lengths of 46 to 70 bp were detected in the mitogenome of *Coccushesperidum* (Table 1), including 15 tRNAs located on the forward strand and the rest on the reverse strand. Only seven tRNAs had the typical cloverleaf secondary structure (*trnD*, *trnL1*, *trnL2*, *trnM*, *trnW*, *trnF* and *trnK*). Of the remaining tRNAs, *trnA*, *trnR*, *trnN*, *trnQ*, *trnS2*, *trnY* and *trnV* lacked the dihydrouridine (DHU) arm, *trnC*, *trnE*, *trnG*, *trnH*, *trnI*, *trnP* and *trnT* lacked the TψC (T) arm and *trnS1* had neither the DHU arm nor the T arm. The majority of tRNAs in our *C.hesperidum* sample used A as the discriminator nucleotide, whereas *trnA*, *trnC*, *trnH*, *trnI*, *trnL1*, *trnS2*, *trnW* and *trnY* used U, *trnD* used C and *trnQ* used G. The length of the amino acid acceptor (AA) arm was consistently 6–7 bp amongst all tRNAs. The anticodon (AC) arm in the majority of tRNAs was 5 bp in length, while it was 4 bp in *trnR*, *trnC*, *trnL2*, *trnW* and *trnV* and 6 bp in *trnT*. Most anticodon loops had nine nucleotides, except for seven nucleotides in *trnC* and 11 nucleotides in *trnV*. The length of the T arm was 2–5 bp and the TψC (T) loop comprised 5–12 nucleotides. The length of the DHU arm was mostly 3 or 4 bp, only in *trnT*, it was 2 bp and *trnE* with 5 bp. The base pairs of tRNAs followed the Watson-Crick pairing rules, while there were eight tRNAs containing a total of 11 G-U pairs with weak attraction (Suppl. material 1: fig. S1).

The two rRNA genes *rrnL* and *rrnS* were both on the reverse strand in the mitogenome of *C.hesperidum*. The *rrnL* gene was 1,174 bp with 87.56% A and T nucleotides and was located between *trnL1* and *trnV*. The *rrnS* gene was 612 bp with an 83.01% A+T content and was located between *trnV* and *trnM* (Table 2).

### ﻿Comparison mitogenomes of scale insects

Mitogenomes of all reported scale insects were compared. All mitogenomes ranged in size from 12,395 bp (*Drosichacorpulenta*, Monophlebidae) to 17,405 bp (*Albotachardinasinensis*, Kerriidae), except for *D.corpulenta* with incomplete annotation information (only base composition and AT-skew were analysed). With respect to the base composition of the whole mitogenomes, the A+T content ranged from 81.0% (*Nipponaclerdabiwakoensis*, Aclerdidae) to 91.1% (*Matsucoccusmatsumurae*, Matsucoccidae), AT-skew ranged from 0.015 (*Phenacoccusmanihoti*, Pseudococcidae) to 0.412 (*Didesmococcuskoreanus*, Coccidae) and GC-skew ranged from -0.573 (*Antecerococcustheydoni*, Cerococcidae) to -0.258 (*D.corpulenta*), indicating that A and T were used more frequently than G and C (Table 4). Using the same method for calculation, the nucleotide compositions of the complete mitogenomes of the 17 scale insects, showed a positive AT-skew and negative GC-skew (Table 4), indicating that the base compositions of most scale insect mitogenomes were biased to A.

**Table 4.** Structural features in the mitogenomes of scale insects.

Family	Species	Mitochondrial genome				PCGs			
		Length	A+T(%)	AT-skew	GC-skew	Length	A+T(%)	AT-skew	GC-skew
Coccidae	*Coccushesperidum*	15566 bp	83.4	0.221	-0.305	10575 bp	82.1	-0.08	-0.11
Coccidae	*Didesmococcuskoreanus*	15143 bp	82.5	0.412	-0.363	10599 bp	81.9	-0.08	-0.10
Coccidae	*Saissetiacoffeae*	15389 bp	84.7	0.190	-0.372	10632 bp	84.1	-0.07	-0.11
Coccidae	*Ceroplastesfloridensis*	15086 bp	85.1	0.231	-0.365	10647 bp	84.5	-0.06	-0.09
Coccidae	*Ceroplastesjaponicus*	14979 bp	85.2	0.232	-0.369	9306 bp	83.7	0.01	-0.15
Coccidae	*Parasaissetianigra*	15632 bp	85.9	0.211	-0.338	10644 bp	85.6	-0.05	-0.07
Coccidae	*Ceroplastesrubens*	15387 bp	87.5	0.241	-0.312	10656 bp	86.6	-0.05	-0.07
Coccidae	*Ericeruspela*	16349 bp	88.4	0.128	-0.316	10659 bp	87.8	-0.06	-0.07
Aclerdidae	*Aclerdatakahashii*	16599 bp	84.5	0.124	-0.444	10608 bp	83.7	-0.10	-0.10
Aclerdidae	*Nipponaclerdabiwakoensis*	16675 bp	81.0	0.128	-0.394	10641 bp	81.0	-0.09	-0.07
Pseudococcidae	*Phenacoccusmanihoti*	14965 bp	89.3	0.015	-0.327	10614 bp	88.8	-0.15	-0.11
Matsucoccidae	*Matsucoccusmatsumurae*	15360 bp	91.1	0.125	-0.305	10623 bp	90.4	-0.12	0.01
Cerococcidae	*Antecerococcustheydoni*	15552 bp	83.6	0.217	-0.573	10584 bp	81.8	-0.07	-0.17
Eriococcidae	*Apiomorphamunita*	15644 bp	89.4	0.031	-0.434	10602 bp	88.1	-0.08	-0.15
Eriococcidae	*Acanthococcuscoriaceus*	16295 bp	89.4	0.087	-0.415	10500 bp	88.0	-0.10	-0.12
Kerriidae	*Albotachardinasinensis*	17405 bp	90.0	0.177	-0.417	10566 bp	89.1	-0.10	-0.16
Monophlebidae	Drosichacorpulenta*	12395 bp	87.2	0.056	-0.258				

*This mitogenome with incomplete annotation information.

**Figure 3.**
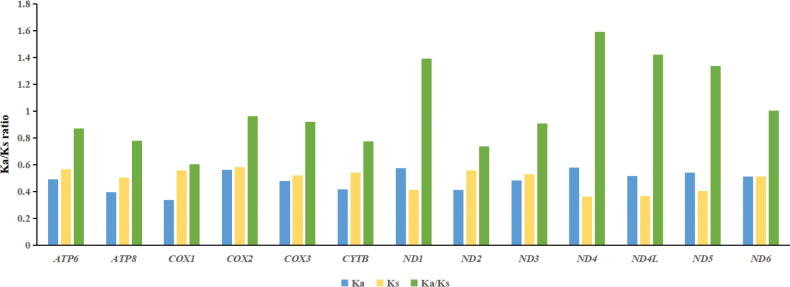
The average Ka/Ks ratios of protein-coding genes (PCGs) in mitogenomes of scale insects.

Within the scale insect mitogenomes, the total length of 13 PCGs ranged from 9,306 bp (*Ceroplastesjaponicus*, Coccidae) to 10,659 bp (*Ericeruspela*, Coccidae), the A+T content and AT/GC skew of each PCG are shown in Table 4 and Suppl. material 1: table S3. All PCGs in the mitogenomes of these scale insects used the typical initiation codon ATN. The majority of PCGs in the species examined ended with a complete and conventional stop codon (TAA or TAG); exceptions included *COX1*, *COX2*, *ND4*, *ND5* and *ND6* genes which terminated with an incomplete stop codon (T). Moreover, RSCU showed obvious bias and different species preferred to use different codons; the most frequently used codons were ATA (Met), ATT (Ile), TTT (Phe) and TTA (Leu) (Suppl. material 1: table S2 and fig. S2). Of the available codons, the most commonly used were all composed of either A or T, consistent with the mitogenome bias towards AT. We further evaluated the evolutionary rates of PCGs (Fig. 3). The Ka/Ks ratios ranged from 0.603 for *COX1* to 1.591 for *ND4*. Amongst the 13 PCGs, values for *ND1*, *ND4*, *ND4L*, *ND5* and *ND6* were greater than 1.0, while values for the others were below 1.0.

The locations of two rRNAs were identical in the majority of scale insect mitogenomes, where *rrnS* was flanked by *trnV* and *trnM* and *rrnL* was flanked by *trnL1* and *trnV*. In other scale insects, the two rRNAs were in different locations; for example, in *M.matsumurae*, *rrnS* was between *trnV* and *trnI*, in *Albotachardinasinensis*, *rrnS* was located between *trnV* and *trnP*, in *Antecerococcustheydoni* and *Acanthococcuscoriaceus*, *rrnL*-*rrnS* was between *trnL1* and *trnQ* and, in *Apiomorphamunita*, *rrnL*-*rrnS* was between *trnV* and *trnY*.

### ﻿Phylogenetic analysis

**Figure 4.**
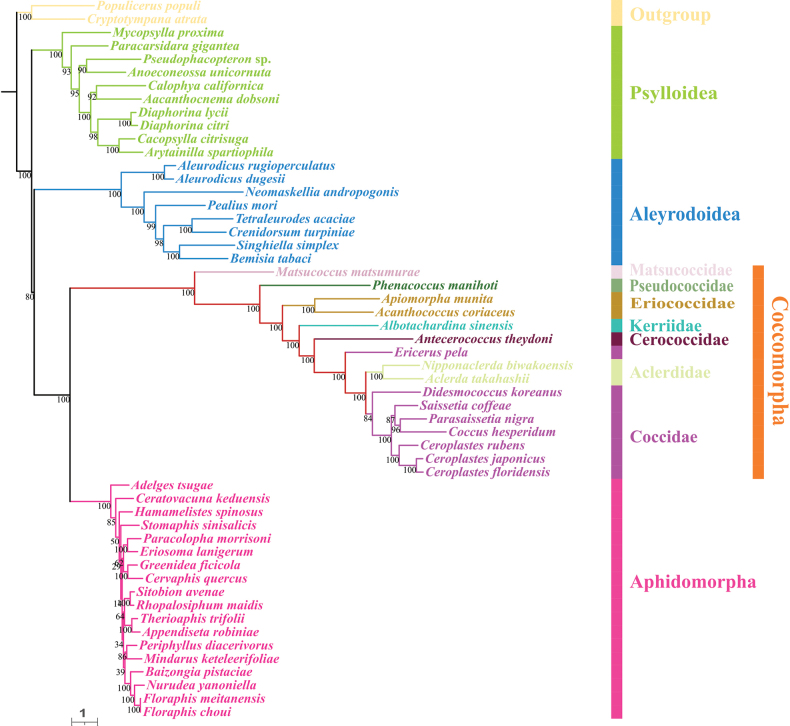
Phylogeny of Sternorrhyncha inferred from the ML tree, based on the PCGAA dataset. Bootstrap support values are indicated at nodes.

**Figure 5.**
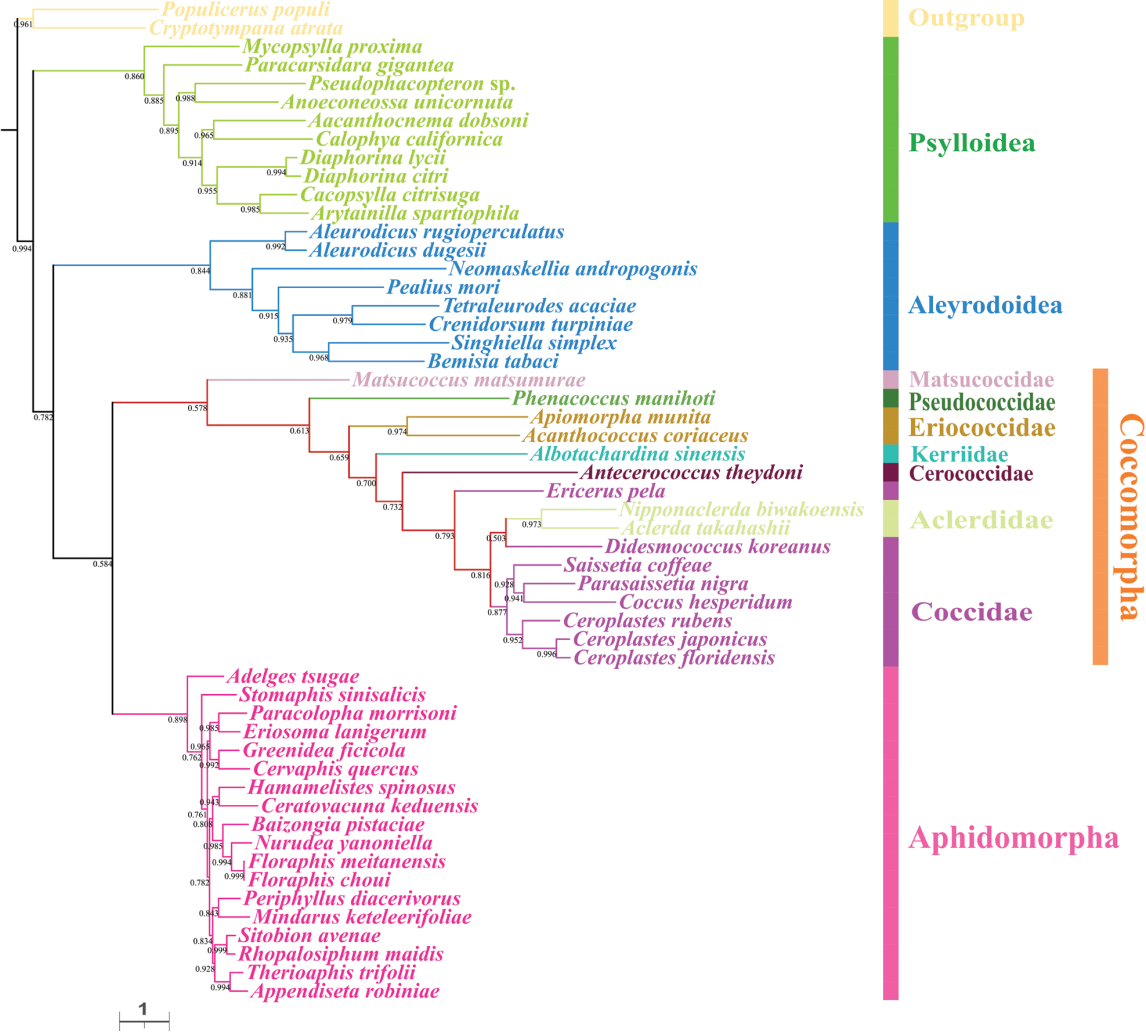
Phylogeny of Sternorrhyncha inferred from the BI tree, based on the PCGAA dataset. Bayesian posterior probabilities are indicated at nodes.

**Figure 6.**
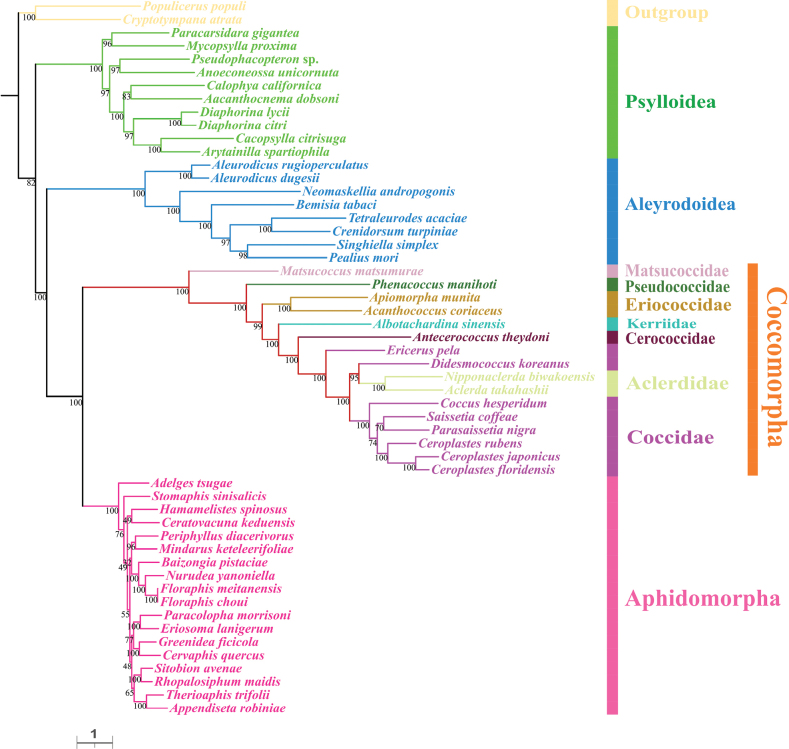
Phylogeny of Sternorrhyncha inferred from the ML tree, based on the PCG123rRNAs dataset. Bootstrap support values are indicated at nodes.

**Figure 7.**
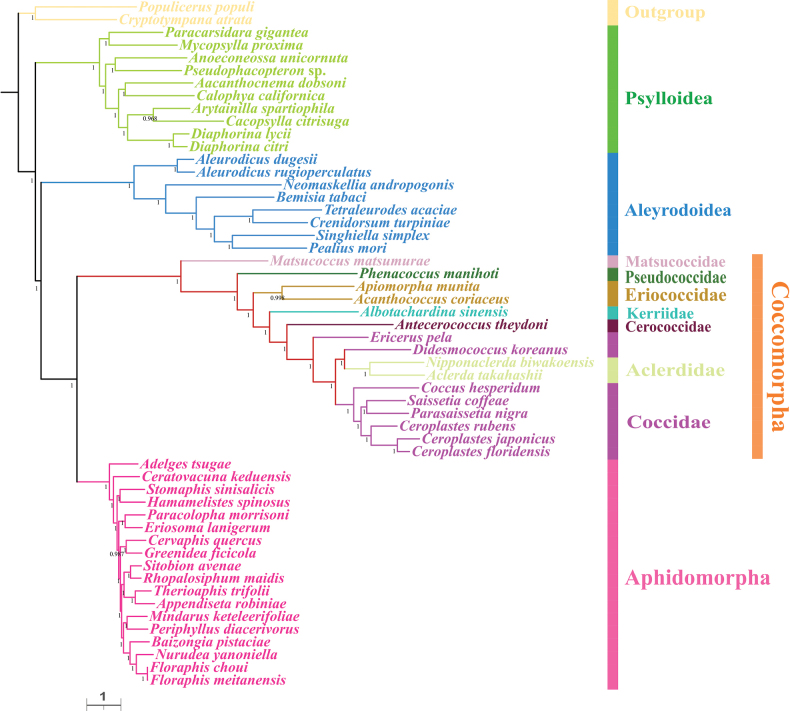
Phylogeny of Sternorrhyncha inferred from the BI tree, based on the PCG123rRNAs dataset. Bayesian posterior probabilities are indicated at nodes.

For each taxon, the PCGAA dataset included 3,127 amino acids and the PCG123rRNA dataset contained 11,380 bp. We obtained four phylogenetic trees with highly concordant topologies, based on the above datasets under BI and ML (Figs 4–7). The trees, based on the PCGAA dataset, were very similar to those based on the PCG123rRNA dataset, especially for the four infraorders or superfamilies Psylloidea, Aleyrodoidea, Aphidomorpha and Coccomorpha, which clustered into monophyletic clades with high support. In all ML and BI analyses, the superfamily Psylloidea was the most basal lineage and as the sister group to the remainder of Sternorrhyncha, Aleyrodoidea was the sister group to a clade composed of Aphidomorpha and Coccomorpha. However, the relationships within each superfamily differed amongst trees, based on different datasets and we focused on phylogenetic relationships amongst scale insects.

Within Coccomorpha, the monophyly of the four families, Pseudococcidae, Matsucoccidae, Cerococcidae and Kerriidae could not be verified because a single species was included for each family. The two families, Eriococcidae and Aclerdidae, represented by two species, formed monophyletic clades with high support. Matsucoccidae, which belonged to the archaeococcoids, was at the most basal position within Coccomorpha. All the remaining families belonging to neococcoids were clustered into a single clade and Pseudococcidae was the basal family of the neococcoids. The phylogenetic relationships of other families belonging to neococcoids were presented as (Eriococcidae + (Kerriidae + (Cerococcidae + (Aclerdidae + Coccidae)))). Thereinto, the two families Aclerdidae and Coccidae were clustered with each other in all phylogenetic trees. However, the species in the family Coccidae did not form a separate clade and were mixed with Aclerdidae species with low to high support values in all BI and ML trees, revealing a surprising/unexpected result that Coccidae appeared as a paraphyletic group and it is necessary to include mitogenomes from more species to confirm this result. Amongst soft scale insects, species in the subfamily Ceroplastinae, represented by the congeneric species *Ceroplastesrubens*, *C.floridensis* and *C.japonicus*, were correctly clustered into one branch with very high support and the species *Coccushesperidum*, *Saissetiacoffeae* and *Parasaissetianigra* of the subfamily Coccinae formed a separate branch in most trees.

## ﻿Discussion

With the development of high-throughput sequencing technology, an increasing number of insect mitogenomes have been sequenced and reported, providing useful data for systematics and evolutionary studies (Pakendorf and Stoneking 2005; Cameron 2014). In the present study, we reported the first mitogenome of *Coccushesperidum*, a species belonging to the tribe Coccini and subfamily Coccinae, increasing the scale insect mitogenomes available in GenBank to 17 species. As in other scale insect mitogenomes, the *C.hesperidum* mitogenome contained a typical set of 37 genes, comprising 13 PCGs, 22 tRNAs and two rRNAs (Lu et al. 2020; Lu et al. 2023). Nucleotide bias is a common phenomenon in the mitogenomes of insects (Cameron 2014; Li et al. 2017; Zhang et al. 2021; Xu et al. 2023). This pattern was detected in the mitogenome of *C.hesperidum*, indicating a significant A+T bias in the mitogenomes of scale insects. Lu et al. (2023) have suggested that the high A+T content of scale insects may be due to evolutionary adaptation to host plants lacking organic nitrogen.

The Ka/Ks ratio is a measure of the selection pressure acting on a gene, indicating neutral selection (Ka/Ks = 1), negative or purifying selection (Ka/Ks < 1) and positive or diversifying selection (Ka/Ks > 1) (Hurst 2002; Luo et al. 2022). A recent study of the evolutionary rates of PCGs in five scale insects has shown that the *ND4L* gene has the highest evolutionary rate, the *COX1* gene had the lowest and nine out of the 13 PCGs show high non-synonymous mutation rates (Ka/Ks > 1) (Lu et al. 2023). Our analysis of 16 scale insects also showed that the *COX1* gene had the lowest evolutionary rate, demonstrating that this gene is conserved relative to other mitochondrial genes, further supporting its use in molecular barcoding in phylogenetic analyses of coccids (Deng et al. 2012; Amouroux et al. 2017; Choi and Lee 2020). However, the results of this study showed that the *ND4* gene had the highest evolutionary rate and five genes had Ka/Ks ratios greater than 1.0. Since our study included data for many species, the results may provide a precise overview of the evolutionary forces shaping scale insect mitogenomes. Of course, the number of mitogenomes of scale insects is still very limited and more mitogenome data are needed to determine the evolutionary rates of scale insects in the future.

Codon usage analyses of the scale insects in our study showed that the most frequently used codons were ATA (Met), ATT (Ile), TTT (Phe) and TTA (Leu) and the least used codons varied amongst species. Furthermore, TAA or TAG was more frequently used as stop codons in most mitogenomes of 16 scale insects, while those in some species ended with a single T. With respect to the secondary structure of tRNAs in the mitogenomes of scale insects, some tRNAs had a typical clover-leaf secondary structure, while some lacked the DHU arm or T arm, forming a truncated secondary structure. A few tRNAs did not have the DHU arm or T arm. Combined with results of previous studies (Lu et al. 2020; Lu et al. 2023; Xu et al. 2023), we speculated that the lack of the DHU arm or T arm might be a common phenomenon in scale insects. tRNAs play an important role in protein synthesis; however, a truncated tRNA does not mean it does not function properly. For example, in nematodes, tRNAs without the DHU and T arms still function normally and these aberrant tRNAs maintain their function through a post-transcriptional RNA editing mechanism (Ojala et al. 1981; Lu et al. 2023). Base pairs of tRNAs generally follow the Watson-Crick pairing rules; however, in addition to the typical A-U and G-C pairing, non-standard pairing was found in scale insects. The most common nucleotide mismatch was G-U, which might play an important role in maintaining the stability of the tRNA secondary structure (Roe et al. 2021; Wu et al. 2022).

Concerning the phylogenetic relationships within Sternorrhyncha, the sister group relationship between Aphidomorpha and Coccomorpha was strongly supported in the present study, congruent with results of previous morphological and molecular studies (Goodchild 1966; Schlee 1969; Boulard 1988; Misof et al. 2014; Johnson et al. 2018; Wang et al. 2019). Psylloidea and Aleyrodoidea have long been controversial and the taxon occupying the most basal position is unclear. Very early studies supported a close sister group relationship between Psylloidea and Aleyrodoidea (Goodchild 1966; Schlee 1969; Boulard 1988), while Johnson et al. (2018) proposed that the deepest divergence within Sternorrhyncha was between Aleyrodoidea and all other taxa based on transcriptomes. However, Song et al. (2019) provided the first mitogenomic data for a Coccomorpha species and proposed that the most basal lineage was Psylloidea and this has been confirmed in some subsequent studies (Lu et al. 2020; Liu et al. 2022; Lu et al. 2023; Xu et al. 2023). Consistent with this, our results showed that Psylloidea, rather than Aleyrodoidea, was the sister group to the remaining taxa in this suborder. Despite this, more data and comprehensive methods are needed to confirm this result. Besides, our results confirmed the sister group relationship between Coccomorpha and Aphidomorpha.

The Coccomorpha is often divided into two informal groups, archaeococcoids and neococcoids. The adult females of the former group possess abdominal spiracles, considered an ancestral feature in scale insects and have been identified as the basal lineage within Coccomorpha (Gullan and Cook 2007; Hodgson and Hardy 2013). Our results showed the species *Matsucoccusmatsumurae* from Matsucoccidae was at the basal position within Coccomorpha, confirming that the archaeococcoids was the earliest lineage of scale insects. Moreover, our phylogenetic analysis showed that the neococcoids (including six families) clustered into a clade and Pseudococcidae was as the basal family of the neococcoids, consistent with previous results (Cook et al. 2002; Gullan and Cook 2007; Hodgson and Hardy 2013; Xu et al. 2023). In addition, the sister group relationship between Aclerdidae and Coccidae has been hypothesised and supported, based on morphological data, DNA sequences and mitogenomes (Hodgson and Hardy 2013; Vea and Grimaldi 2016; Xu et al. 2023). However, our results showed that *Ericeruspela* and *Didesmococcuskoreanus*, belonging to Eulecaniinae of Coccidae, were often assigned to clades including Aclerdidae, rendering Coccidae paraphyletic. Similar results were obtained in a BI tree, based on the PCGrRNA dataset by Xu et al. (Xu et al. 2023). Owing to a lack of data availability, taxon sampling of Coccomorpha species, based on mitogenomes, is still very limited; thus, increasing the sample size for this group is expected to clarify the relationships within Coccomorpha.

## ﻿Conclusions

The present study is the first to determine the complete mitogenome sequence of *Coccushesperidum* (tribe Coccini) by next-generation sequencing methods. The *C.hesperidum* mitogenome was 15,566 bp long, had a high A+T content (83.4%) and contained a typical set of 37 genes, with 13 PCGs, 22 tRNAs and two rRNAs. Only seven tRNAs had the typical cloverleaf secondary structure and the remaining tRNAs lacked the DHU arm, TψC arm or both. The mitogenomes of all reported scale insects were similar in structure, base composition and A+T content. As determined by RSCU, there was obvious bias and different coccid species preferred to use different codons; the most frequently used codons were ATA (Met), ATT (Ile), TTT (Phe) and TTA (Leu). Our phylogenetic analysis confirmed the monophyly of Coccomorpha, demonstrated that the archaeococcoids occupied the most basal position within Coccomorpha and showed that *Ericeruspela* and *Didesmococcuskoreanus*, belonging to Coccidae, were mixed with Aclerdidae, such that Coccidae may form a paraphyletic group. Collectively, this study enriches the mitogenome database of scale insects and provides the basis for future phylogenetic and evolutionary analyses of scale insects.

## ﻿Additional information

### Author ORCIDs

Yun-Feng Hou https://orcid.org/0009-0008-8244-7389

Tian-You Zhao https://orcid.org/0000-0003-1378-6893

Fang Wang https://orcid.org/0000-0002-5758-7434

### Data availability

All of the data that support the findings of this study are available in the main text or Supplementary Information.

## Supplementary materials

Supplementary material 1 First complete mitochondrial genome of the tribe Coccini and its phylogenetic implications Data type zip Explanation note **table S1**. List of species used in the phylogenetic analysis. **table S2.** The most frequently used codons of protein-coding genes (PCGs) in scale insect mitogenomes. **figure S1.** Inferred secondary structures of 22 transfer RNA genes (tRNAs) of *Coccushesperidum*. tRNAs are labelled with abbreviations for the corresponding amino acids according to the IUPAC-IUB code. **table S3.** A+T content (%) in mitogenomes of scale insects. **figure S2**. The relative synonymous codon usage (RSCU) of protein-coding genes (PCGs) in mitogenomes of scale insects. https://binary.pensoft.net/file/911967This dataset is made available under the Open Database License (http://opendatacommons.org/licenses/odbl/1.0/). The Open Database License (ODbL) is a license agreement intended to allow users to freely share, modify, and use this Dataset while maintaining this same freedom for others, provided that the original source and author(s) are credited.Yun-Feng Hou, Jiu-Feng Wei, Tian-You Zhao, Cai-Feng Li, Fang Wang
