# Supplementary material for: ﻿First complete mitochondrial genome of the tribe Coccini (Hemiptera, Coccomorpha, Coccidae) and its phylogenetic implications
Source: Zookeys. 2023 Sep 26;1180:333–54. doi: 10.3897/zookeys.1180.109116 (PMC10838174; doi:10.3897/zookeys.1180.109116)
Supplement: Supplementary material 1 — First complete mitochondrial genome of the tribe Coccini and its phylogenetic implications [file zookeys-1180-333_article-109116__-s001.zip › 109116_1C-1-A_revised_Supplementary_Material_1_Table_S1._List_of_species_used_in_the_phylogenetic_analysis.docx]

**Table S1.** List of species used in the phylogenetic analysis.

| Suborder | Infraorder/Superfamily | Family | Species | GenBank ID |
| --- | --- | --- | --- | --- |
| Sternorrhyncha | Coccomorpha | Coccidae | *Coccus hesperidum* | OR167606 |
|  |  | Coccidae | *Parasaissetia nigra* | OK040656 |
|  |  | Coccidae | *Ceroplastes rubens* | OP388828 |
|  |  | Coccidae | *Didesmococcus koreanus* | NC057479 |
|  |  | Coccidae | *Ceroplastes floridensis* | NC067791 |
|  |  | Coccidae | *Ceroplastes japonicus* | MK847519 |
|  |  | Coccidae | *Saissetia coffeae* | MN863803 |
|  |  | Coccidae | *Ericerus pela* | OP388829 |
|  |  | Pseudococcidae | *Phenacoccus manihoti* | NC066716 |
|  |  | Matsucoccidae | *Matsucoccus matsumurae* | NC070232 |
|  |  | Aclerdidae | *Aclerda takahashii* | MW839575 |
|  |  | Aclerdidae | *Nipponaclerda biwakoensis* | OP351526 |
|  |  | Eriococcidae | *Apiomorpha munita* | OP351523 |
|  |  | Eriococcidae | *Acanthococcus coriaceus* | OP351525 |
|  |  | Kerridae | *Albotachardina sinensis* | OP351521 |
|  |  | Cerococcidea | *Antecerococcus theydoni* | OP351522 |
|  | Aleyrodoidea | Aleyrodidae | *Crenidorsum turpiniae* | NC050930 |
|  |  | Aleyrodidae | *Neomaskellia andropogonis* | NC006159 |
|  |  | Aleyrodidae | *Pealius mori* | LR877884 |
|  |  | Aleyrodidae | *Singhiella simplex* | LR877885 |
|  |  | Aleyrodidae | *Tetraleurodes acaciae* | NC006292 |
|  |  | Aleyrodidae | *Aleurodicus rugioperculatus* | MW649000 |
|  |  | Aleyrodidae | *Aleurodicus dugesii* | NC005939 |
|  |  | Aleyrodidae | *Bemisia tabaci* | KY951452 |
|  | Aphidomorpha | Aphididae | *Rhopalosiphum maidis* | OR148359 |
|  |  | Aphididae | *Stomaphis sinisalicis* | NC053790 |
|  |  | Aphididae | *Sitobion avenae* | NC024683 |
|  |  | Aphididae | *Greenidea ficicola* | OM350400 |
|  |  | Aphididae | *Cervaphis quercus* | NC024926 |
|  |  | Aphididae | *Periphyllus diacerivorus* | MZ665537 |
|  |  | Aphididae | *Therioaphis trifolii* | MK766411 |
|  |  | Aphididae | *Appendiseta robiniae* | MH643884 |
|  |  | Hormaphididae | *Ceratovacuna keduensis* | NC063091 |
|  |  | Hormaphididae | *Hamamelistes spinosus* | NC050942 |
|  |  | Pemphigidae | *Floraphis choui* | NC035310 |
|  |  | Pemphigidae | *Floraphis meitanensis* | NC035316 |
|  |  | Pemphigidae | *Nurudea yanoniella* | NC035313 |
|  |  | Pemphigidae | *Baizongia pistaciae* | NC035314 |
|  |  | Pemphigidae | *Paracolopha morrisoni* | NC045103 |
|  |  | Pemphigidae | *Eriosoma lanigerum* | NC033352 |
|  |  | Mindaridae | *Mindarus keteleerifoliae* | KP722576 |
|  |  | Adelgoidae | *Adelges tsugae* | MT263947 |
|  | Psylloidea | Aphalaridae | *Anoeconeossa unicornuta* | NC038108 |
|  |  | Calophyidae | *Calophya californica* | NC036302 |
|  |  | Carsidaridae | *Paracarsidara gigantea* | NC038112 |
|  |  | Homotomidae | *Mycopsylla proxima* | NC037225 |
|  |  | Liviidae | *Diaphorina citri* | NC030214 |
|  |  | Liviidae | *Diaphorina lycii* | NC036352 |
|  |  | Phacopteronidae | *Pseudophacopteron* sp. | MG989234 |
|  |  | Psyllidae | *Arytainilla spartiophila* | NC038133 |
|  |  | Psyllidae | *Cacopsylla citrisuga* | NC053749 |
|  |  | Triozidae | *Aacanthocnema dobsoni* | NC038132 |
| Auchenorrhyncha | Cicadoidea | Cicadidae | *Cryptotympana atrata* | NC058223 |
|  |  | Cicadellidae | *Populicerus populi* | NC039427 |
