# Supplementary material for: ﻿First complete mitochondrial genome of the tribe Coccini (Hemiptera, Coccomorpha, Coccidae) and its phylogenetic implications
Source: Zookeys. 2023 Sep 26;1180:333–54. doi: 10.3897/zookeys.1180.109116 (PMC10838174; doi:10.3897/zookeys.1180.109116)
Supplement: Supplementary material 1 — First complete mitochondrial genome of the tribe Coccini and its phylogenetic implications [file zookeys-1180-333_article-109116__-s001.zip › 109116_1C-1-A_revised_Supplementary_Material_2_Table_S2._The_most_frequently_used_codons_of_protein_coding_genes_(PCGs)_in_scale_insect_mitogenomes.docx]

**Table S3.** The most frequently used codons of protein coding genes (PCGs) in scale insect mitogenomes.

| Family | Species | ATA | TTT | ATT | TTA |
| --- | --- | --- | --- | --- | --- |
| Coccidae | *Coccus hesperidum* | 463 | 413 | 324 |  |
| Coccidae | *Ceroplastes floridensis* | 558 | 451 | 357 |  |
| Coccidae | *Ceroplastes japonicus* | 533 | 313 | 286 |  |
| Coccidae | *Ceroplastes rubens* | 636 | 475 | 362 |  |
| Coccidae | *Didesmococcus koreanus* | 419 | 383 | 357 |  |
| Coccidae | *Ericerus pela* | 606 | 416 | 428 |  |
| Coccidae | *Parasaissetia nigra* | 571 | 431 | 370 |  |
| Coccidae | *Saissetia coffeae* | 509 | 423 | 338 |  |
| Aclerdidae | *Aclerda takahashii* | 438 | 390 | 405 |  |
| Aclerdidae | *Nipponaclerda biwakoensis* | 400 |  | 382 | 337 |
| Cerococcidae | *Antecerococcus theydoni* | 424 | 405 | 344 |  |
| Eriococcidae | *Acanthococcus coriaceus* | 447 |  | 510 | 462 |
| Eriococcidae | *Apiomorpha munita* | 488 |  | 478 | 465 |
| Kerriidae | *Albotachardina sinensis* | 420 |  | 569 | 502 |
| Matsucoccidae | *Matsucoccus matsumurae* | 488 |  | 497 | 509 |
| Pseudococcidae | *Phenacoccus manihoti* |  | 499 | 500 | 474 |
