# Supplementary material for: ﻿First complete mitochondrial genome of the tribe Coccini (Hemiptera, Coccomorpha, Coccidae) and its phylogenetic implications
Source: Zookeys. 2023 Sep 26;1180:333–54. doi: 10.3897/zookeys.1180.109116 (PMC10838174; doi:10.3897/zookeys.1180.109116)
Supplement: Supplementary material 1 — First complete mitochondrial genome of the tribe Coccini and its phylogenetic implications [file zookeys-1180-333_article-109116__-s001.zip › 109116_1C-1-A_revised_Supplementary_Material_4_Table_S3._A+T_content_(%)_in_mitogenomes_of_scale_insects.docx]

Table S3. A+T content (%) in mitogenomes of scale insects.

|  | *ATP6* | *ATP8* | *COX1* | *COX2* | *COX3* | *CYTB* | *ND1* | *ND2* | *ND3* | *ND4* | *ND4L* | *ND5* | *ND6* |
| --- | --- | --- | --- | --- | --- | --- | --- | --- | --- | --- | --- | --- | --- |
| *Coccus hesperidum* | 83.8 | 91.5 | 75.8 | 78.6 | 82.0 | 78.7 | 81.9 | 84.7 | 86.5 | 84.6 | 88.2 | 83.7 | 87.6 |
| *Didesmococcus koreanus* | 83.7 | 85.8 | 75.6 | 79.9 | 83.9 | 80.3 | 82.2 | 83.1 | 81.3 | 83.3 | 86.2 | 84.2 | 86.0 |
| *Saissetia coffeae* | 84.7 | 85.1 | 77.0 | 81.4 | 84.5 | 81.8 | 83.6 | 88.1 | 87.2 | 86.8 | 89.8 | 86.2 | 87.7 |
| *Ceroplastes floridensis* | 84.9 | 86.5 | 77.8 | 80.4 | 85.0 | 82.3 | 85.2 | 88.3 | 87.2 | 86.9 | 90.1 | 85.4 | 89.6 |
| *Ceroplastes japonicus* | 84.5 | 87.9 | 76.6 | 81.6 | 84.7 | 82.1 | 84.4 | 88.7 | 87.1 | 86.0 | 89.6 | 82.2 | 91.2 |
| *Ceroplastes rubens* | 89.0 | 88.7 | 79.9 | 83.2 | 85.7 | 83.9 | 85.8 | 90.8 | 90.2 | 88.8 | 90.8 | 88.6 | 91.0 |
| *Parasaissetia nigra* | 88.2 | 90.0 | 78.3 | 80.4 | 86.5 | 83.4 | 94.3 | 89.4 | 90.0 | 86.8 | 89.7 | 87.9 | 92.3 |
| *Ericerus pela* | 89.1 | 90.7 | 81.7 | 85.4 | 88.4 | 85.8 | 87.4 | 90.7 | 89.5 | 89.7 | 89.2 | 89.6 | 93.5 |
| *Aclerda takahashii* | 84.2 | 88.0 | 75.8 | 83.6 | 84.1 | 81.5 | 83.5 | 86.5 | 88.4 | 85.5 | 88.5 | 84.9 | 90.5 |
| *Nipponaclerda biwakoensis* | 80.8 | 86.1 | 73.9 | 77.1 | 81.9 | 79.0 | 78.4 | 85.5 | 81.5 | 83.2 | 83.7 | 81.8 | 85.3 |
| *Phenacoccus manihoti* | 90.2 | 86.3 | 80.4 | 85.8 | 90.5 | 87.6 | 89.6 | 93.1 | 89.6 | 92.1 | 95.2 | 89.6 | 93.3 |
| *Matsucoccus matsumurae* | 92.8 | 91.6 | 85.0 | 88.6 | 91.5 | 88.3 | 88.2 | 95.3 | 93.2 | 91.1 | 96.1 | 90.8 | 94.6 |
| *Antecerococcus theydoni* | 81.5 | 84.0 | 74.8 | 78.7 | 81.3 | 79.4 | 84.1 | 84.0 | 85.7 | 83.8 | 86.3 | 83.9 | 88.6 |
| *Apiomorpha munita* | 87.7 | 91.4 | 79.8 | 84.9 | 88.6 | 86.4 | 89.1 | 91.6 | 89.1 | 91.3 | 94.7 | 90.6 | 92.6 |
| *Acanthococcus coriaceus* | 89.7 | 91.5 | 79.7 | 86.0 | 89.2 | 85.5 | 87.4 | 92.0 | 90.2 | 90.2 | 93.3 | 89.8 | 95.5 |
| *Albotachardina sinensis* | 90.7 | 91.4 | 82.2 | 85.4 | 88.2 | 86.8 | 89.9 | 91.9 | 92.6 | 92.6 | 95.8 | 90.7 | 92.0 |
