# Supplementary material for: ﻿First complete mitochondrial genome of the tribe Coccini (Hemiptera, Coccomorpha, Coccidae) and its phylogenetic implications
Source: Zookeys. 2023 Sep 26;1180:333–54. doi: 10.3897/zookeys.1180.109116 (PMC10838174; doi:10.3897/zookeys.1180.109116)
Supplement: Supplementary material 1 — First complete mitochondrial genome of the tribe Coccini and its phylogenetic implications [file zookeys-1180-333_article-109116__-s001.zip › Figure legends.docx]

Figure S1. Inferred secondary structures of 22 transfer RNA genes (tRNAs) of *Coccus hesperidum.* tRNAs are labeled with abbreviations for the corresponding amino acids according to the IUPAC-IUB code.

Figure S2. The relative synonymous codon usage (RSCU) of protein-coding genes (PCGs) in mitogenomes of scale insects.
